# Optical Spin Hall Effect in Closed Elliptical Plasmonic Nanoslit with Noncircular Symmetry

**DOI:** 10.3390/nano11040851

**Published:** 2021-03-26

**Authors:** Xiaorong Ren, Xiangyu Zeng, Chunxiang Liu, Chuanfu Cheng, Ruirui Zhang, Yuqin Zhang, Zijun Zhan, Qian Kong, Rui Sun, Chen Cheng

**Affiliations:** 1 College of Physics and Electronics, Shandong Normal University, Jinan 250014, China; bidud@126.com (X.R.); zengxiangyu0611@163.com (X.Z.); liuchunxiang@sdnu.edu.cn (C.L.); zhangruirui0268@163.com (R.Z.); ss_yghg@163.com (Y.Z.); zhanzijun1990@163.com (Z.Z.); kongqian0304@163.com (Q.K.); sunrui199812@163.com (R.S.); 2 School of Electronic and Information Engineering, Qilu University of Technology (Shandong Academy of Sciences), Jinan 250353, China

**Keywords:** surface plasmons, optical spin Hall effect, geometric phases, phase gradient

## Abstract

We investigated the optical spin Hall effect (OSHE) of the light field from a closed elliptical metallic curvilinear nanoslit instead of the usual truncated curvilinear nanoslit. By making use of the characteristic bright spots in the light field formed by the noncircular symmetry of the elliptical slit and by introducing a method to separate the incident spin component (ISC) and converted spin component (CSC) of the output field, the OSHE manifested in the spot shifts in the CSC was more clearly observable and easily measurable. The slope of the elliptical slit, which was inverse along the principal axes, provided a geometric phase gradient to yield the opposite shifts of the characteristic spots in centrosymmetry, with a double shift achieved between the spots. Regarding the mechanism of this phenomenon, the flip of the spin angular momentum (SAM) of CSC gave rise to an extrinsic orbital angular momentum corresponding to the shifts of the wavelet profiles of slit elements in the same rotational direction to satisfy the conservation law. The analytical calculation and simulation of finite-difference time domain were performed for both the slit element and the whole slit ellipse, and the evolutions of the spot shifts as well as the underlying OSHE with the parameters of the ellipse were achieved. Experimental demonstrations were conducted and had consistent results. This study could be of great significance for subjects related to the applications of the OSHE.

## 1. Introduction

The optical spin Hall effect (OSHE), a phenomenon originally demonstrated by the spin-dependent transverse shifts of light reflected or refracted by a plane interface, has attracted enormous interest and evoked many pioneering studies [1,2,3,4,5,6] since it was proposed by Onoda as an analogy to the electron spin Hall effect [7]. Inherently, the OSHE is a manifestation of the geometric phase in light propagation [8,9], which is originated from the spin–orbit interactions (SOIs) of light [1,3] and has provided a novel route for the spin-dependent manipulation of light field, such as generalized refraction [1], holography with metasurfaces [10,11,12], and creation of specific beams or fields [13,14,15,16].

The OSHE is related to two types of geometric phases—the Rytov–Vladimirskii–Berry (RVB) phase [8,17,18] and the Pancharatnam–Berry (PB) phase [9,19,20]. The former is imposed on a light wave while its propagating direction is changed. It typically occurs when light is reflected and refracted at a medium interface that is sharply inhomogeneous but isotropic, where each angular spectral component in the light wave acquires a different RVB phase, thereby forming an RVB phase gradient and providing a spin-dependent real-space split of the light beam perpendicular to the plane of incidence. This split is a direct demonstration of the OSHE but its small magnitude has been a challenge to measurements, for which different judicious schemes have been proposed such as weak measurements [21,22,23] and multiple reflections of light in a cylindrical glass rod [24]. The PB phase is related to the change in the polarization state of light, which may occur when light propagates in anisotropic and inhomogeneous systems. Although the spatial variant PB phase sets a phase gradient, it yields spin-dependent split of light waves and gives rise to OSHE in momentum space. The shift between the light waves in different spin components increases with the propagation of light, which renders the direct observation and measurement of OSHE much easier and simpler. Furthermore, in current nanophotonics, the manipulation of the PB phase has become the basic method in the design of artificial metamaterials, in which the spatial distributions of the light fields are flexibly and conveniently controlled by polarization variations, thereby providing typical examples for the application of OSHE and SOIs [25,26,27,28,29,30]. The functionality of the PB phase and the spin Hall effect in the nanostructure design of metamaterials have demonstrated wide perspectives in compact and miniaturized engineering of nanophotonic devices, which is of great importance for the development of optical communication, precision metrology, quantum information processing, and so forth. [4,26,27,28,29,30].

Bliokh et al. proposed a semicircular metallic slit as the focusing plasmonic nanostructure to demonstrate the OSHE [18]; this has overcome difficulties and inaccuracies in observing and measuring small shifts in large-sized light fields in the previous observations of the effect in reflection and refraction at medium interfaces. Thereafter, plasmonic curvilinear nanostructures have attracted great interest for investigating the characteristics of OSHE in different regimes. Until now, various curvilinear nanostructures, such as catenary slits [31], plasmonic chains [32], and PB phase metasurfaces [3,4,10,11,12,13,14,15,16], have been used for applications. The key feature of nanostructures is their noncircular symmetry, which is usually achieved by truncating a closed curvilinear nanoslit in such a way that the spatially asymmetric shift of the spin-dependent light wavefield takes place in the direction perpendicular to the axis of reflection symmetry of the structure. In regimes of slit structures of closed curves with noncircular but reflection symmetry, the OSHE and the transverse shifts in the directions of the reflection axis may occur, and novel characteristics of the light field may be achieved correspondingly; however, they have rarely been studied in such structures.

It is clear that, for nanoslits of smaller width, the SOIs of light with the slits change the polarization states of light under circular polarization (CP) illumination [33], and the output field generally contains the two CP components of opposite helicities. The one with the same helicity as the incident light is referred to as incident spin component (ISC), and the other with the opposite helicity is referred to as converted spin component (CSC), which acquires a PB phase shift twice the local orientation angle of the nanoslit. The OSHE occurs in the light field of the CSC owing to the acquired PB phase. Nevertheless, in previous studies on the OSHE in curvilinear slits, the two components in output fields have been analyzed as a whole without distinguishing them based on whether the effect occurs or not. Obviously, with the two components separated and the nonexistence of OSHE in ISC being considered, the characteristics of the transverse shifts and the nature of OSHE occurring in such regimes could be better comprehended.

In this work, the OSHE in closed curvilinear nanoslit of centrosymmetry in metallic films was investigated using an elliptical nanoslit as the sample. Using the characteristic spots in the light field formed by the elliptical slit breaking the circular symmetry and by introducing a method to separate the CSC and the ISC of the output field, the characteristic shifts of the spots in the CSC reflecting the OSHE were clearly observable and easily measurable. The shifts could be obtained quantitatively either by comparing the spots in CSC with the corresponding unshifted spots in ISC or by comparing a centrosymmetric pair of counterpart spots in the CSC field that were shifted in opposite directions. The varied tangent slope of the curvilinear ellipse provided a geometric phase gradient in the CSC field, which gave rise to shifts in characteristic spots demonstrating the OSHE. The slope inversion in the reflection symmetric parts of the ellipse about the principal axes offered inverse geometric phase gradients, thereby causing the opposite shifts of the characteristic spots in centrosymmetry with the shift between them doubled. To compensate for the flip of the spin angular momentum (SAM), an extrinsic orbital angular momentum (OAM) was generated by an element of the slit ellipse, while the shifts for the two elements of reflection symmetry were in the same rotational direction around the ellipse circumference, and the intersection point of the profiles shifted in the direction perpendicular to the axis of reflection symmetry. Moreover, when the observation plane moved farther from the surface of the metallic slit, the geometric phase gradient resulted in the transverse component of the momentum, thereby yielding the linearly increased shifts of characteristic spots with greater observation distance. A slower increase in the shifts was demonstrated in the direction of the major axis because of the smaller geometric phase gradient compared to that of the minor axis. The feasibility of the proposed method and the results were analytically and experimentally demonstrated. The present work provides a new nanostructure regime and route for investigating the OSHE.

## 2. Theoretical Analysis of OSHE Produced by an Elliptical Curvilinear Nanoslit

### 2.1. Plasmonic Wave Field Based on the Incident and Converted Spin Components

**Figure 1:**
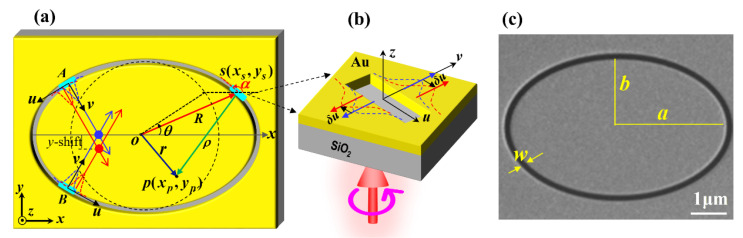
Schematic of a slit element of the elliptical gold nanoslit embedded on silica substrate. (**a**) Top view of the elliptical nanoslit and the basic geometric parameters. (**b**) Magnified demonstration of the shifts of the surface plasmon polaritons produced by an element. (**c**) Scanning electron microscope image of elliptical nanoslit etched using focused ion beam.

An elliptical curvilinear nanoslit in gold film on a silica substrate is depicted in Figure 1; Figure 1a is a schematic of the nanoslit, Figure 1b is the magnified demonstration of a slit element, and Figure 1c presents the scanning electron microscope image of a practical sample. The width of the elliptical nanoslit is *w*, and the semimajor and semiminor axes of the inner edge are *a* and *b*, respectively, as shown in Figure 1c. In the Cartesian coordinate system, the ellipse is expressed by *x*^2^/*a*^2^ + *y*^2^/*b*^2^ = 1, and the corresponding parametric representation is *x* = *a∙*cos*θ* and *y* = *b∙*sin*θ* with 0 ≤ *θ* ≤ 2π, as shown in Figure 1a. The light field ***E***(*x_p_*, *y_p_*) at a point *p*(*x_p_*, *y_p_*) on the film surface is the superposition of the light fields excited by all the slit elements of the elliptical nanoslit with a continuously varying orientation angle *α*. The element at the point *s*(*x_s_*, *y_s_*) in Figure 1b acts as an individual scatterer of surface plasmon polariton (SPP) and can be treated as a birefringent wave plate [34] with the Jones matrix.

(1) $$J(\alpha )=R(-\alpha )\left[\begin{array}{cc} t_{u} & 0\\ 0 & t_{v} \end{array}\right]R(\alpha ),$$

where *t_u_* = |*t_u_*|∙exp(*iφ_u_*) and *t_v_* = |*t_v_*|∙exp(*iφ_v_*) denote the transmission coefficients along the two orthogonal principal axes, and *R*(.) is the rotation matrix.

When the nanoslit is illuminated by CP light $E_{in}^{\sigma }$ = [1 − *σi*]*^T^*/$\sqrt{2}$ propagating along the *z*-axis, the excited SPP wave field is linearly polarized (LP) in the direction perpendicular to the nanoslit element, as shown in Figure 1a,b, and the corresponding coefficients are *t_u_* = 1 and *t_v_* = 1. Consequently, the output SPP wave is written as:

(2) $$E(x_{s},\;y_{s})=J(\alpha )E_{in}^{\sigma }=\frac{1}{2\sqrt{2}}\left[\begin{array}{c} 1\\ \sigma i \end{array}\right]+\frac{1}{2\sqrt{2}}\left[\begin{array}{c} 1\\ -\sigma i \end{array}\right]\exp(i2\sigma \alpha ).$$

Here, *σ* = ±1 denotes the right- and left-handed circular polarization (RCP and LCP), respectively. Obviously, the right side of Equation (2) includes two terms. The first one is referred to as ISC, which has the same helicity as the incident light, and is denoted by ***E****^σσ^*(*x_s_*, *y_s_*) = [1 − *σi*]*^T^*/2√2; the second one is CSC, which is denoted by ***E****^σ^*^−*σ*^(*x_s_*, *y_s_*) = [1 − *σi*]*^T^* exp(−*i*2*σα*)/2√2 and has an helicity opposite to the incident light. It can be noticed that the CSC acquires a spin-dependent geometric phase Φ = 2*σα*, which is a phase shift achieved in the cyclic transformation of the polarization state and corresponds to a closed loop circuit on the Poincaré sphere in the geometry of the parameter space [9,35,36,37].

The slit element d*l* at point *s*(*x_s_*, *y_s_*) on the ellipse is regarded as a radiating source, and the output wave ***E***(*x_s_*, *y_s_*)d*l* can be obtained based on Equation (2). Hence, the wave field it produces at point *p*(*x_p_*, *y_p_*) near the center of the ellipse can be written as [38]

(3) $$dE(x_{p},y_{p})=-(i/\sqrt{\lambda_{spp}})\;dl\cos\gamma E(x_{s},y_{s})\exp(ik_{spp}\rho )\exp(i\pi /4)/\sqrt{\rho }$$

where *ρ* is the distance between *p* and *s*, cos*γ* the inclination factor, and *k_spp_ =* 2π*/λ_spp_* the wave vector of the SPPs. From the geometry shown in Figure 1a, we get *ρ =* |***R*** *– **r**|*, where |***R****| =* (*a*^2^cos^2^*θ + b*^2^sin^2^*θ*)^1/2^ is the radial distance from point *s* on the ellipse to the origin. The infinitesimal length of the source can be expressed as d*l* = (*a*^2^sin^2^*θ + b*^2^cos^2^*θ*)^1/2^ d*θ = R’*d*θ*. Thus, the wave field excited by the whole elliptical nanoslit can be calculated as the integral along the closed trajectory of the ellipse.

(4) $$E(x_{p},y_{p})=C\;\int_{0}^{2\pi }d\theta R{}^{\prime }\cos\gamma E(x_{s},y_{s})\exp(ik_{spp}\rho )/\sqrt{\rho },$$

where *C* is a complex constant. Substituting Equation (2) into Equation (4), the wave field ***E***(*x_p_*,*y_p_*) at point *p*(*x_p_*, *y_p_*) can be written as

(5) $$E\left(x_{p},y_{p}\right)\;=E^{\sigma \sigma }\left(x_{p},y_{p}\right)+E^{\sigma -\sigma }\left(x_{p},y_{p}\right),$$

with

(6a) $$E^{\sigma \sigma }(x_{p},y_{p})=C{}^{\prime }\int_{0}^{2\pi }d\theta R{}^{\prime }\cos\gamma \left[\begin{array}{c} 1\\ \sigma i \end{array}\right]\exp(ik_{spp}\rho )/\sqrt{\rho }$$

(6b) $$E^{\sigma -\sigma }(x_{p},y_{p})=C{}^{\prime }\int_{0}^{2\pi }d\theta R{}^{\prime }\cos\gamma \left[\begin{array}{c} 1\\ -\sigma i \end{array}\right]\exp(ik_{spp}\rho +\Phi )/\sqrt{\rho }.$$

Here*, **E**^σσ^*(*x_p_*, *y_p_*) and ***E**^σ-σ^*(*x_p_*, *y_p_*) are the ISC and CSC of ***E*** (*x_p_*, *y_p_*), respectively. Equations (6a) and (6b) indicate that the wave field ***E*** (*x_p_*, *y_p_*) indeed contains the ISC ***E**^σσ^*(*x_p_*, *y_p_*) and CSC ***E**^σ-σ^*(*x_p_*, *y_p_*) of CP. Because the additional geometric phase Φ = 2*σα* in Equations (6a) and (6b) varies along the ellipse with the variant tangential angle *α*, a nonuniform gradient of this phase will arise, thereby resulting in a change in the wave vector ***k*** of the CSC relative to the ISC. Thus, the CSC will experience a change in propagating directions and a variation of spatial distribution, and when characteristic intensity distributions such as bright or dark spots exist in the wave field, the spots in the CSC will separate from those in the ISC. As a result, the two component fields of orthogonal circular polarization will split, which is a typical manifestation of the OSHE.

The wave field of Equation (2) can equivalently be expressed as the superposition of two orthogonal LP components ***E***(*x_s_*, *y_s_*) = *E_x_*(*x_s_*, *y_s_*)$\hat{x}$+ *E_y_* (*x_s_*, *y_s_*)$\hat{y}$ with the unit direction vector $\left(\hat{x},\hat{y}\right)$. Therefore, the LP components are:

(7) $$\left[\begin{array}{c} E_{x}\left(x_{s},y_{s}\right)\\ E_{y}\left(x_{s},y_{s}\right) \end{array}\right]=\left[\begin{array}{c} 1\\ \sigma i \end{array}\right]+\left[\begin{array}{c} 1\\ -\sigma i \end{array}\right]\exp(i\Phi ).$$

The wave field ***E***(*x_p_*, *y_p_*) in the CP components ***E**^σσ^*(*x_p_*, *y_p_*) and ***E****^σ-σ^*(*x_p_*, *y_p_*) of Equations (6a) and (6b) can also be written directly as Cartesian coordinate components as follows:

(8a) $$E_{x}(x_{p},y_{p})=\int_{0}^{2\pi }d\theta R{}^{\prime }\cos\gamma [1+\exp(i\Phi )]\exp(ik_{spp}\rho )/\sqrt{\rho }$$

(8b) $$E_{y}(x_{p},y_{p})=\int_{0}^{2\pi }d\theta R{}^{\prime }\cos\gamma [1-\exp(i\Phi )]\sigma i\exp(ik_{spp}\rho )/\sqrt{\rho }.$$

The above equations indicate that each of the LP components *E_x_* (*x_p_*, *y_p_*) and *E_y_* (*x_p_*, *y_p_*) of the field ***E***(*x_p_*, *y_p_*) is the addition of the corresponding components in the ISC and CSC, with CSC being imposed on the geometric phase. Owing to the shift in the CSC intensity distribution related to this phase, the superposition of ISC and CSC may spatially average the shift of the component fields *E_x_*(*x_p_*, *y_p_*) and *E_y_*(*x_p_*, *y_p_*). This results in a diminished displacement in the LP component fields in comparison with that in CSC. 

### 2.2. Characteristic Spot Shifts Depending on the Geometric Phase Gradient 

The direct solutions of light fields in the above equations are unachievable for elliptical slit, but based on these equations, the light field shifts due to the geometric phase can be analyzed physically and numerically. The gradient of the phase of the light field is related to the wave vector ***k***, which also represents the photon momentum. Thereupon, the geometric phase gradient ∇Φ from the elliptical slit provides an additional transverse component Δ***k****^σ^* in the momentum *k* of CSC and is directly written as [39]

(9) $$\Delta k^{\sigma }=\nabla \Phi =\partial_{x}\Phi \;\hat{x}+\partial_{y}\Phi \;\hat{y},$$

where *∂_x_* and *∂_y_* denote the derivatives with respect to *x* and *y*, respectively. With the tangential angle *α* = *π*/2 − arctan[*a*^2^*x*/(*b*^2^*y*)], the derivative of Φ is simply expressed as *∂_x_*_(*y*)_Φ = 2*σ∂_x_*_(*y*)_∙*α*. Thus, the geometric phase gradients of the CSC in parameter representation are obtained.

(10a) $$\partial_{x}\Phi =-2\sigma b\sin\theta /(a^{2}\sin^{2}\theta +b^{2}\cos^{2}\theta )$$

(10b) $$\partial_{y}\Phi =2\sigma a\cos\theta /(a^{2}\sin^{2}\theta +b^{2}\cos^{2}\theta ).$$

It follows that:

(11) $$\Delta k^{\sigma }=[-2\sigma b\sin\theta /(a^{2}\sin^{2}\theta +b^{2}\cos^{2}\theta )]\hat{x}+[2\sigma a\cos\theta /(a^{2}\sin^{2}\theta +b^{2}\cos^{2}\theta )]\hat{y}$$

The influence of the additional momentum Δ***k****^σ^* on the propagation and consequently on the spatial distribution of the light field is analyzed in two aspects. First, in the gold–air interface, the change in the propagating direction due to the momentum change Δ***k****^σ^* in the wavelet excited by a slit element at azimuth *θ* causes the superposed field of all the wavelets to exhibit the whole-field in-plane shifts in the interface. Second, for the light distributions at a transverse plane away from the interface, Δ***k****^σ^* provides an additional transverse deflection for the wavelet from a slit element and thus gives rise to additional transverse shifts $\delta_{ex}^{\left(z\right)}$ and $\delta_{ey}^{\left(z\right)}$ increased with distance *z* linearly, with the subscript as the notion of a slit element. The shift of the light field exiting from the element includes two parts: the initial shifts corresponding to *δ_x_* and *δ_y_* at the interface, and $\delta_{ex}^{\left(z\right)}$ and $\delta_{ey}^{\left(z\right)}$ increasing with *z* by $\delta_{e}^{\left(z\right)}$*=* Δ*k^σ^∙z*/*k*_0_ *=* $\delta_{ex}^{\left(z\right)}$$\hat{x}$+ $\delta_{ey}^{\left(z\right)}$***ŷ*** [39]. From Equation (11), we get:

(12a) $$\delta_{ex}^{\left(z\right)}=[-2\sigma b\sin\theta /(a^{2}\sin^{2}\theta +b^{2}\cos^{2}\theta )](\lambda_{0}/\pi )z$$

(12b) $$\delta_{ey}^{\left(z\right)}=[2\sigma a\cos\theta /(a^{2}\sin^{2}\theta +b^{2}\cos^{2}\theta )](\lambda_{0}/\pi )z.$$

**Figure 2:**
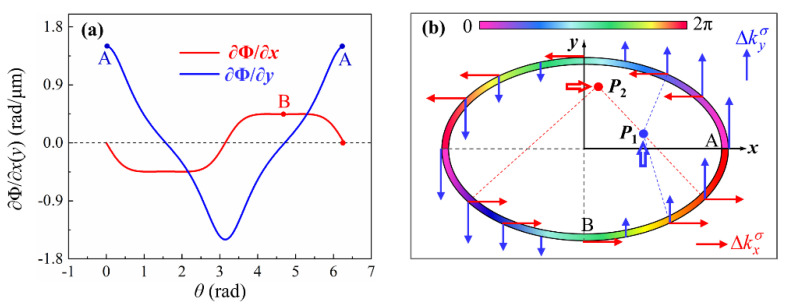
(**a**) Geometric phase gradients at different positions (different *θ*) on the elliptical nanoslit. (**b**) Schematic diagram demonstrating the shifts of the characteristic spots *P*_1_ and *P*_2_ due to geometric phase gradients ∂*_y_*Φ and ∂*_x_*Φ, respectively; the color bar represents the geometric phase.

To comprehend the overall effect of the phase gradient ∇Φ on the shift of light intensity distributions and the characteristic spots, we plotted the curves of gradients *∂_x_* Φ and *∂_y_*Φ along the elliptical nanoslit as a function of azimuth *θ* in Figure 2a, which are calculated based on Equations (10a) and (10b). The basic characteristics of the curves for *∂_x_*Φ and *∂_y_*Φ are noticeable. In a comparatively large range of *θ*, the curve of ∂*_x_*Φ remains nearly negative and positive constant values in the intervals [0, π] and [π, 2π], respectively, corresponding to the upper and lower halves of the ellipse, respectively, with the corresponding momentum demonstrated by the red arrows on the ellipse in Figure 2b. The curve of *∂_y_*Φ has positive and negative values in the intervals [–*π*/2, π/2] and [π/2, 3π/2], respectively, corresponding to the right and left halves of the ellipse, respectively; on both sides of the midpoint of each interval, the curve increases and decreases linearly symmetrically, and the momentum is indicated by the blue arrows in Figure 2b.

Subsequently, as an example, we observe the most obvious characteristic spot *P*_1_ focused by the right half of the elliptical slit, which yields the phase gradient *∂_y_*Φ in the upward direction, as indicated in Figure 2b. The gradient *∂_y_*Φ, as the additional momentum component, is added to the primary momentum of the wavelet from the element on the elliptical slit and causes the propagation direction of the wavelet to change upward. As a result, the characteristic spot *P*_1_, as the overall superposition of the wavelets from the right half of the ellipse, shifts upward; this is demonstrated in Figure 2b and can be observed in the following sections. Similarly, for the characteristic spot *P*_2_ formed by the focusing of the lower half of the ellipse, the additional momentum component corresponding to the phase gradient *∂_x_*Φ in the rightward direction is added to the primary momentum of the wavelet from the slit, thereby causing the propagation direction of the wavelet to change rightward and the spot *P*_2_ to shift rightward thereupon. Furthermore, the phase gradient on either the left or the upper half of the ellipse causes the shift of the spot in a direction opposite to that of the counterpart spot *P*_1_ or spot *P*_2_, respectively. 

Based on the above analysis, it can be inferred that, in contrast to OSHE in semicircular slit with a single spot shifting along the diameter, the OSHE in the elliptical slit exhibits the opposite shifts in pairs of counterpart spots along the major and minor axes, respectively, and thus the effect becomes more obvious and more easily observable.

## 3. Simulations and Discussions

To illustrate the OSHE in the elliptical nanoslit, we first performed simulations using the finite-difference time domain (FDTD) as an accurate numerical method to achieve the light field. The thickness of the gold film was 200 nm, and the parameters of the elliptical nanoslit shown in Figure 1c were *a* = 3 µm, *b* = 2 µm, and *w* = 100 nm. The CP light with a wavelength of 632.8 nm illuminated the sample at the normal incidence. For comparison and physical analysis, we also conducted numerical integral calculations of the wave fields of the elliptical nanoslit based on Equations (6a)–(8b). In the FDTD performance, the simulation area was set to 6.4 µm × 5 µm × 4 µm, with a minimum mesh cell size of 5 nm. We proposed an algorithm that numerically mimicked a polarization filter composed of a quarter-wave plate and a polarizer, through which LCP and RCP fields are separated and ISC and CSC are obtained. In the numerical integral calculations, the CP components ***E****^σσ^*(*x_p_*, *y_p_*) and ***E****^σ-σ^*(*x_p_*, *y_p_*) as well as the LP components ***E****_x_*(*x_p_*, *y_p_*) and ***E****_y_*(*x_p_*, *y_p_*) of the wave field distributions at the interface *z* = 0 were calculated directly.

### 3.1. Centrosymmetric Spot Shifts of OSHE Related to the Noncircular Symmetry of Elliptical Slit

**Figure 3:**
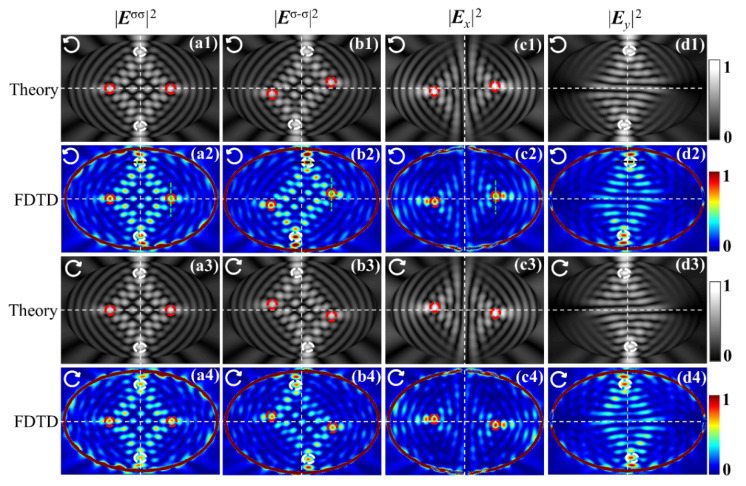
(**a1**–**d4**) Intensity maps of different components at the gold–air interface *z* = 0 obtained by finite-difference time domain (FDTD) simulation (rows 2 and 4) and numerical integral calculations based on Equations (6a)–(8b) (rows 1 and 3) for left-handed circular polarization (LCP) (rows 1 and 2) and right-handed circular polarization (RCP) (rows 3 and 4) illuminations. In the columns from left to right, the intensity maps are for the incident spin component, converted spin component (CSC), *x*-component, and *y*-component, respectively. The incidences are denoted by the white arrows.

Unlike the semicircular nanoslit with noncircular symmetry but reflection symmetry about the central axis perpendicular to the diameter, the elliptical nanoslit is a structure with noncircular symmetry but reflection symmetry about the *y*- and *x*-axes simultaneously, thereby leading to the geometric phase gradients in both *x-* and *y*-directions, respectively. Thus, the characteristic spots shifting in the *x*-direction (white circles) and others shifting in the *y*-direction (red circles) occur simultaneously in transmission images, as shown in Figure 3(b1–d4). Figure 3 depicts the intensity images of the ISC, CSC, *x*-component, and *y*-component of the transmitted light fields obtained with the numerical integral calculations based on Equations (6a)–(8b) as well as FDTD at the gold–air interface *z* = 0 under the illumination of the RCP and LCP, respectively. The white dashed horizontal and vertical lines are drawn to designate the *x*- and *y*-axis, respectively. The ISC and CSC intensity maps inside the ellipse exhibit bright spot-like distributions originating from the reflection but noncircular symmetry, that is, reflection symmetry about the *x*- and *y*-axis but asymmetry under a rotation from the *x*-axis to the *y*-axis of the elliptical nanoslit. The bright spots with high intensities can be regarded as the characteristic spots, which are marked with circles. On the one hand, for the maps of the ISC, the distributions of the light field along with the included bright and characteristic spots are reflection symmetric about the *x*- and *y*-axis, which derives from the superposition of wavelets from the elliptical slit of reflection symmetry. On the other hand, in the images of the CSC, each characteristic spot shifts either vertically or horizontally relative to its counterpart in the ICS image, depending on the locations on either the *x*- or *y*-axis.

Moreover, because of the centrosymmetry of the elliptical slit, the intensity distributions of the CSC are also centrosymmetric; therefore, they appear as coinciding bright spot distributions as a 180° rotation about the *z*-axis of the ellipse center is accomplished. Furthermore, the shifts of the characteristic spots in a symmetrical pair are equal but opposite with respect to the *x*- or *y*-axis, and the separation between a pair of spots is twice the shift of each spot. Physically, this results from the noncircular but reflection symmetry of the ellipse, and under such a symmetry, the slope of the ellipse and the corresponding gradient of the additional geometric phase are inverse with respect to the *x*- or *y*-axis, thereby giving rise to the opposite shift of the characteristic spots and the centrosymmetric intensity distributions, as described above. In each of the intensity maps of the LP components ***E****_x_*(*x_p_, y_p_*) and ***E****_y_*(*x_p_*, *y_p_*), there are only two centrosymmetric characteristic spots, marked with red or white circles in Figure 3, in contrast to the four characteristic spots in two symmetric pairs distributed along the *x*-axis or *y*-axis, respectively, in the ISC and CSC maps. Interestingly, the two spots in the *x*-polarized component are distributed along the *x*-axis, but their shifts are opposite in the *y*-direction, and the same is true for the spots in the *y*-polarized component with their shifts in the *x*-direction. This means that the spilt of the characteristic spots is perpendicular to the direction of the linear polarization. When the closed elliptical nanoslit is used to measure the OSHE, it might be advantageous over the previous truncated semicircle nanoslit. In the semicircular nanoslit system, the shift of the single spot from the center of the semicircle is used, and the unobservable center as the reference point needs to be accurately determined, which would bring about inconvenience for the performance and additional errors of measurement. While in the elliptical nanoslit system, the relative shift between the characteristic spots in a symmetrical pair is doubled, the symmetric spots are clearly observable and the shift is determined without needs for other reference; this makes the OSHE more easily measurable with better accuracy.

**Figure 4:**
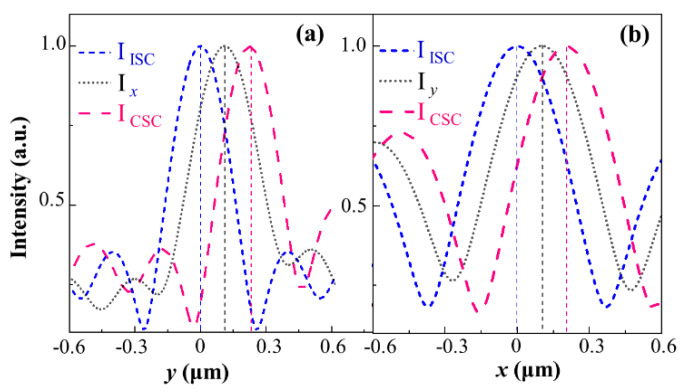
Normalized intensity profile curves of the characteristic bright spots extracted from Figure 3(a2,b2,c2) along the green vertical dash lines (**a**) and from Figure 3(a2,b2,d2) along the black horizontal dash lines (**b**). The distributions in all curves are normalized to their maximum values. Different colors refer to different components.

Obviously, the shifts between the characteristic spots in the *x*- and *y*-component fields are smaller than (actually half) those between the spots of the corresponding symmetric pair in the CSC field, as shown in Figure 3, with the spots in corresponding pairs marked with red and white circles, respectively, for clarity. This happens because the fields of the *x*- and *y*-component are the addition of the fields of corresponding component in both CSC and ISC, as noticed in Equations (8a) and (8b), and the shifts of spots occur solely in the CSC field, not in the ISC field. To analyze the shifts more quantitatively, the intensity curves through the centers of the characteristic bright spots were extracted from the images of the *x*- and *y*-component, together with the curves through the corresponding spots from CSC and ISC, respectively, are shown in Figure 4. The curves in Figure 4a designate the intensity data along the vertical lines, marked with the dashed green vertical line segments in Figure 2(a2–c2), while the curves in Figure 4b indicate the data along the horizontal lines, marked with the black segments in Figure 3(a2,b2,d2). For the curves in Figure 4a, the *y*-coordinates corresponding to the peaks of the intensity curves for the *x*-component and CSC are practically the spot shifts along the *y*-direction, referred to as *y*-shifts, which are read at 0.1098 µm and 0.2218 µm, respectively, under the parameter values set for the simulations; meanwhile, the peak at zero *y*-coordinate in the ISC intensity curve can be used as the reference for the shifts of the corresponding spots in the *x*-component and in the CSC fields. 

Similarly, in Figure 4b, the shifts along the *x*-direction, referred to as *x*-shifts, of the characteristic spots in the *y*-component, CSC and ISC, are 0.1032 µm, 0.2023 µm, and 0 µm, respectively. These values demonstrate that, with good accuracy, the shifts of the bright spots marked with colored circles in the *x*- and *y*-components are half those of the corresponding spots in the same color circles in the CSC. Because the LP *x*- and *y*-components are the addition of the corresponding components in both ISC and CSC, and the characteristic spots in CSC merely experience a shift relative to their unshifted counterparts in ISC, with their size and brightness almost unchanged, the peaks in the curves of the *x-* and *y*-components are formed at the midpoint of the ISC and CSC peaks, as demonstrated by the obtained shift values. Therefore, the phenomenon in which the shifts of the spots in the LP components are half those in CSC is essentially the result of OSHE occurring solely in the CSC field of the elliptical nanoslit. Although the shifts of the LP component spots have been used to measure the OSHE in curvilinear nanoslit regimes in previous literature, the shifts of the CSC spots, acquired by separating the CSC from the total light field, can be observed more clearly and can demonstrate the mechanisms of the OSHE more explicitly.

It is worth noting that when change in the wavelength *λ* of the illuminating light takes place, the propagation phase shift will change by 2π*s*/*λ* with s being the path difference; as a result, for the intensity distributions of ISC ***E***^σσ^ similar to those in Figure 3, the size of the bright spots and their separation distances will vary with *λ*, but the basic form of the symmetrical intensity distribution remains unchanged. Moreover, the geometric phase depends only on the orientation of the slit element and is independent of wavelength, and correspondingly, the properties of the characteristic spot shifts and their symmetry will also remain basically unchanged.

In addition, by using the FDTD simulations, the efficiency of the elliptical nanoslit for the OSHE were estimated. By taking a rectangle which was matched to include the nanoslit ellipse, the input power was calculated as product of the illuminating light intensity and area of the rectangle; the output power was calculated by integrating the intensity of the transmitted CSC component immediately after the elliptical slit. Thus, we obtained the estimated efficiency 4.45% as the ratio of the output power to the input power. The low efficiency is resulted from the small area ratio of the nanoslit and loss of the metal medium. Fortunately, the transmitted intensity distributions with the characteristic spots are easily measurable in the experimental setup, as will be demonstrated in the later experimental Section.

### 3.2. Conservation of Angular Momentum in SOIs with the Slit

Furthermore, the OSHE is associated with the phenomena of optical SOIs. As the incident light passes through the nanoslit, the change of the polarization state corresponds to the variation of the SAM, which gives rise to a change in the OAM with the conservation of the total angular momentum (TAM). Afterward, the change in the momentum or the propagation direction of light can occur correspondingly. Particularly, regarding RCP light as an example for the incident light with a TAM of ⟨*S*_in_⟩ ∝ 1, while the TAM ⟨*J*⟩ of the transmitted light field is conserved, both the TAMs of CSC ⟨*J*_c_⟩ and ISC ⟨*J*_i_⟩ are conserved as well. Here, the angle brackets ⟨.⟩ represent the mean SAM of the light field [40]. The CSC is flipped to LCP with SAM ⟨*S*_c_⟩ ∝ −1, and the change of SAM is ⟨Δ***S***_c_⟩ = ⟨***S***_c_⟩ − ⟨***S***_in_⟩ ∝ −⟨***P***_c_⟩*/*⟨*P*_c_⟩ − ⟨***P***_in_⟩*/*⟨*P*_in_⟩, where ⟨***P***_in_⟩ and ⟨***P***_c_⟩ are the mean momenta of incident light and CSC, respectively [40]. The conservation of angular momentum ⟨*J*_c_ ⟩ = ⟨*J*_in_⟩ sets ⟨*J*_c_⟩ = ⟨*L*_c_^ext^ ⟩ + ⟨*S*_c_⟩ ∝ 1, with the superscript denoting extrinsic OAM [8,40,41]. The process indicates that ⟨Δ***S*_c_**⟩ introduces a change in extrinsic OAM and equivalently causes changes in light propagations and distributions. One of the possible results to compensate for the flip of the SAM by creating extrinsic OAM in CSC is that the wavelet profile on each side of a slit, as schematically demonstrated by the red dashed curves in Figure 1b, undergoes the transverse shift *δ****u*** or -*δ****u*** along the *u*-axis with respect to an unshifted profile in the blue dashed curve, which represents the profile of the ISC field. The extrinsic OAM of the wavelet propagating in the *v*–direction is accordingly ⟨***L***_c_^ext^ ⟩ = *δ****u*** × ⟨***P***_c_⟩ [40]. For two slit elements A and B, which are reflection symmetric with respect to the *x*-axis, the counterclockwise shifts *δ**u*** of their CSC field profiles correspond to equal extrinsic OAM change and causes the intersection point of their propagating field profiles to shift downward, as shown in Figure 1a, and thus the characteristic spot shifts in the *y*-direction. This schematic is also consistent with the above analysis with the geometric phase gradients. Though somewhat rough, the schematic is simple and intuitive, and more accurate results can be demonstrated by the analytical and numerical integrals and calculations.

### 3.3. Variation of the Spots and the Shifts with the Observation Distance

**Figure 5:**
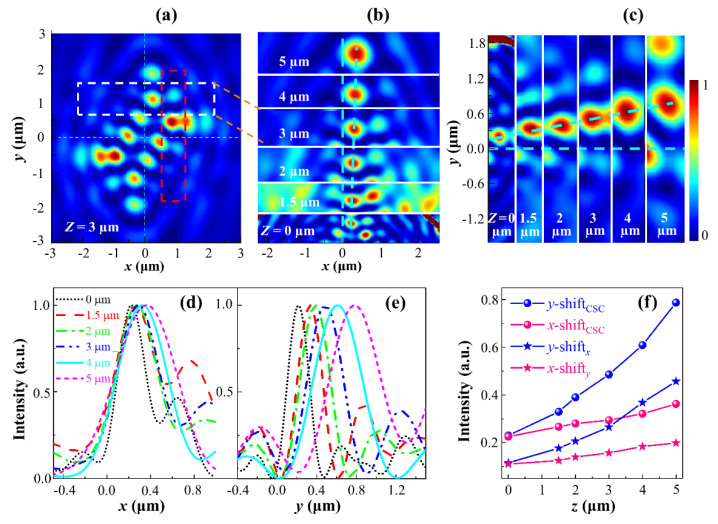
(**a**) Intensity maps of the converted spin component (CSC) light field of *z* = 3 µm with RCP illumination, (**b**–**c**) Intensity stripes including the characteristic spots with *x*-shift (**b**) and *y*-shift (**c**) as the stripes in the white and red dotted boxes in (**a**), respectively, which are intercepted from different intensity maps of *z* = 0–5 µm, (**d**–**e**) Normalized cross-sectional intensity profiles of the characteristic spots in (**b**–**c**), respectively. The distributions in all curves are normalized to their maximum values. Different colors refer to different propagation distances *z*. (**f**) *x*-shifts and *y*-shifts of the characteristic spots in CSC fields (**b**–**c**), and corresponding spots in *x*- and *y*-component fields, respectively.

When the observation plane moves away from the sample surface with distance *z*, the OSHE is manifested as an increase in the characteristic spot shifts in the outgoing CSC component field, as described above. Figure 5a illustrates the full intensity image of the CSC fields obtained with FDTD at *z* = 3 μm, and Figure 5b presents the image consisting of striped intensity images, including the characteristic spots of the *x*-shift at distances from *z* = 0 to *z* = 5 μm, where the striped map at *z* = 3 μm cut off from its full map is marked with the white dashed rectangle in Figure 5a. Figure 5c depicts the striped maps including the characteristic spots of the *y*-shift, and Figure 5a indicates the striped map at *z* = 3 μm marked with the red dashed rectangle. In Figure 5, we can notice that both the *x*- and *y*-shifts of the characteristic spots increase with the propagation distance *z*, which is in good agreement with the dependence of the shift on the propagation distance provided by Equations (10a) and (10b).

For a more quantitative demonstration, Figure 5d–e show the curves of CSC intensity profiles versus *x* and *y*, respectively, passing through the maximum intensity points of characteristic spots along the *y-* and *x*-axis at different distances. The *x-* and *y*-shifts of the spots along the *y*- and *x*-axis are read from the profiles, respectively, and the curves of the *x*- and *y*-shifts versus distance *z* for CSC are illustrated in Figure 5f. Similarly, the curves of the shifts versus distance *z* for *y*- and *x*-polarization components, respectively, are also shown in Figure 5f. From these curves, we notice that the *y*-shift increases more quickly than the *x*-shift for either the CSC or the LP components, as it can be observed more clearly in Figure 5c in comparison with Figure 5b for CSC. We also notice that even the spot size increases more quickly in Figure 5c. This originates from the different distributions of geometrical phase gradients along the *x*- and *y*-directions; the larger deflections in the *y*-direction result from the larger phase gradient, which is consistent with the results of Equations (12a) and (12b) as well as the analysis presented above.

## 4. Experiment

**Figure 6:**
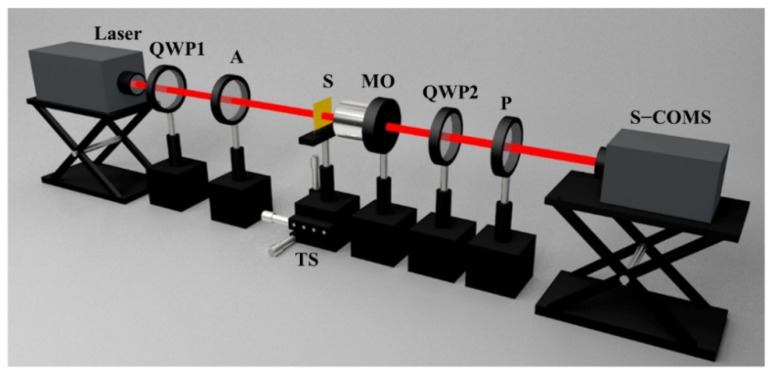
Schematic illustration of the experimental setup. QWP1 and QWP2 represent quarter-wave plates, A is attenuator, S stands for sample, microscope objective lens is denoted by MO, and P is Glan–Thompson polarizer. Laser is He–Ne with an emission wavelength of 632.8 nm, and the S-CMOS camera model is Zyla 5.5.

To verify the OSHE through the closed curvilinear nanoslit, we fabricated a sample of elliptical nanoslits using focused ion beam etching and conducted experimental observations and measurements. The physical parameters of the elliptical nanoslit were the same as those involved in the simulation. A schematic of the experimental setup is illustrated in Figure 6. A He–Ne LP laser of wavelength 632.8 nm with power 30 mw is converted into CP light by a properly oriented quarter-wave plate (QWP1) and is utilized as the incident light to illuminate the sample from the substrate side. The sample was mounted on a three-dimensional transitional stage. The light waves scattered from the sample surface were collected and imaged by a microscope objective (Nikon, NA 0.9, 100×). The light fields of the ISC and the CSC are selected by adjusting QWP2 and polarizer P, and the images are received by an S-CMOS (Zyla 5.5, 16-bit, 2560 × 2160 pixels, pixel size 6.5 μm × 6.5 μm). When QWP2 is removed, by adjusting the transmitting direction of P, the images of the *x*- and *y*-components are recorded.

**Figure 7:**
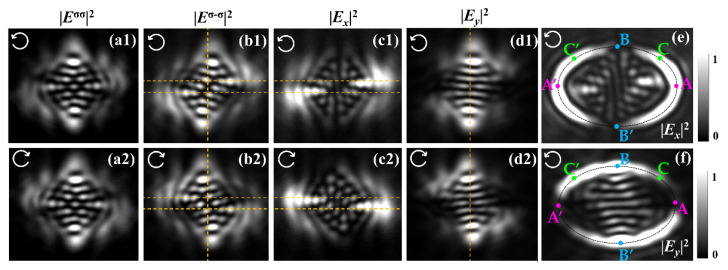
(**a1**–**d2**) Experimental results of different components of transmitted fields with RCP (**a1**–**d1**) and LCP (**a2**–**d2**) illumination at *z* = 2 μm. (**e**–**f**) Experimental results of |***E****_x_*|^2^ and |***E****_y_*|^2^ near the gold–air interface under RCP illumination. The incidences are marked with white arrows.

Figure 7 presents the experimental results for |***E****_x_*|^2^,|***E****_y_*|^2^,|***E****^σσ^*|^2^, and |***E****^σ^*^−*σ*^|^2^ produced by the elliptical nanoslit at a distance *z* = 2 μm, respectively, with the images in Figure 7(a1–d1) in the upper row for RCP illumination and Figure 7(a2–d2) in the lower row for LCP illumination; Figure 7e–f are results for |***E****_x_*|^2^ and |***E****_y_*|^2^ near the gold–air interface for RCP illumination, respectively, where the elliptical slits are marked with dotted lines, points A and A’ are the vertices, and points B and B’ are the co-vertices of the ellipse. The centrosymmetric distributions of characteristic spots in the intensity maps of |***E****_x_*|^2^,|***E****_y_*|^2^, and |***E****^σ^*^−*σ*^|^2^ are obvious, and the centrosymmetric shifts of characteristic spots in the CSC images in Figure 7(b1–b2) with respect to those in ISC images in Figure 7(a1–a2) are the direct experimental verifications of the OSHE.

As can be observed by comparing the corresponding spots in the intensity maps of Figure 7(b1,b2), the shifts of characteristic spots in the CSC images under RCP and LCP illuminations are in opposite directions. This indicates that the geometric phase gradient depends on the spin, as expected from the previous analysis. Similarly, the corresponding centrosymmetric pairs of spots in the intensity maps of |***E****_x_*|^2^ and |***E****_y_*|^2^ shift in opposite directions as well, but the distances between the spots along the *x*- and *y*-axis are smaller than (approximately half) the corresponding distances in the images of |***E****^σ-σ^*|^2^. For clarity in determining the shifts, we designate the characteristic spot pair by drawing two yellow horizontal dashed lines passing through the center of the spots of the *y*-shift in Figure 7(b1–c2) and draw the yellow vertical dashed line to designate the *y*-axis in CSC and |***E****_y_*|^2^ images, respectively. These experimental results were consistent with those obtained by the FDTD simulation shown in Figure 3.

From the maps in Figure 7e,f we can demonstrate the geometric phase imposed on the transmitted wave and slope of the elliptical slit, which was inverse along the principal axes. In Figure 7e, intensity of |***E****_x_*|^2^ vary along the elliptical slit marked by the dotted curve, it takes the maximum values at the vertices A and A,’ and it takes minimum values at the co-vertices B and B.’ Based on Equation (7), *E_x_* ~1 + exp(*i*Φ) is obtained, and the |***E****_x_*|^2^ map is actually the interference of the CSC and the ISC; maximum and minimum intensities at A and B indicate Φ_A_ = 0 and Φ_B_ = π, respectively; similarly, the Φ_A_’ = 2π and Φ_B_’ = 3π are obtained. We now consider the two points C and C’ which are symmetric with respect to minor axis, and geometric phases have also symmetrical values with respect to that of point B, that is, Φ_C_ = π − ΔΦ and Φ_C_ ’= *π* + ΔΦ, respectively. With Δ*α* = ΔΦ/2, we have *α*_C_ = π/2 − ΔΦ/2 and *α*_C_’= π/2 + ΔΦ/2, and consequently, the slopes of points C and C’ are k_C_ = cot(△Φ/2) and k_C_’ = − cot(△Φ/2), respectively, demonstrating that the slope is inverse along the minor axis of the ellipse slit. Similarly, by using *E_y_* ~*i*[1 − exp(*i*Φ)], the same conclusion can be obtained using the intensity of |***E****_y_*|^2^ in Figure 7f, and it can also be demonstrated that the slope of slit is inverse along the major axis of the ellipse slit.

**Figure 8:**
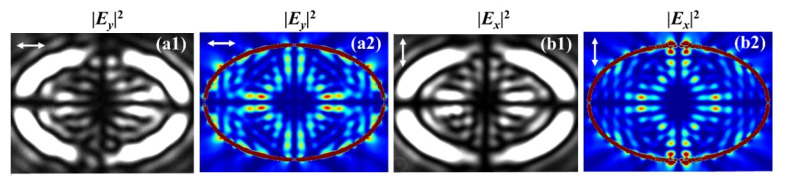
Experimental (**a1**,**b1**) and simulation (**a2**,**b2**) intensity maps of |***E****_y_*|^2^ under the *x*-linearly polarized (LP) illumination (**a1**,**a2**) and |*E_x_*|^2^ under the *y*-LP illumination (**b1**,**b2**), respectively.

Figure 8 depicts the experimental and FDTD results near the gold–air interface with LP light illumination. As the superposition of the RCP and LCP, the LP incident light causes the double shifts of the characteristic bright spots in opposite directions for the transmitted field, and the intensity maps are reflection-symmetric, as can be noticed in Figure 8. For the converted RCP and LCP components, the acquired additional geometric phases are in opposite signs, thus the two different CP component fields split, which is the manifestation of the OSHE. Owing to the noncircular symmetry of the elliptical nanoslit and the subsequent geometric phase gradient in the *x*- and *y*-directions, such splits occur in both the *x*- and *y*-directions simultaneously. Overall, experimental observations demonstrate theoretical analysis and numerical calculations and suggest that the geometric phase associated with the spin–orbit interaction underpins the shifts of the characteristic spots and the OSHE in the closed curvilinear nanoslits.

## 5. Conclusions

In summary, instead of the usual truncated curvilinear nanoslit, a single closed elliptical nanoslit was used to demonstrate and observe the OSHE. Compared to the truncated symmetry-breaking curvilinear slits in literature, the present work employed a closed elliptical slit with the noncircular but reflection symmetry and achieved centrosymmetric shifts occurring as bright characteristic spot pairs in the light field. Instead of the conventional measurement of OSHE in the curvilinear slit regimes in which the two spin components were not distinguished with incomplete understanding of the inherent physics, the polarization separation method was firstly introduced in the investigation, and the transmitted light field was divided into ISC and CSC fields. Thus the shifts of characteristic spots were more clearly observable and the mechanism of the related OSHE was more explicit. The elliptical nanoslit offered the geometric phase gradients of the CSC field, which were inverse about the principal axes; consequently, the characteristic spot pairs shifted in opposite directions and thus the shift between them was doubled. Moreover, the change in the extrinsic OAM resulted from the flip of the SAM in the CSC field and gave rise to the shifts of the wavelet profiles from the slit elements in the same rotational direction, thereby yielding centrosymmetric shifts in the characteristic spot pair. The shifts of the characteristic spots increased with the observation distance linearly, and the smaller phase gradient along the major axis set a slower increase. The principles and results based on the analytical and simulation performances as well as the experimental demonstrations might be developed to different kinds of curvilinear nanoslits for OSHE. We believe that this study could be significant for subjects involving applications of the OSHE, such as nanophotonics, precision metrology and spin-based photonics.

## Data Availability

Data is contained within the article.
